# Bio- and Hemo-Compatible Silk Fibroin PEGylated Nanocarriers for 5-Fluorouracil Chemotherapy in Colorectal Cancer: In Vitro Studies

**DOI:** 10.3390/pharmaceutics13050755

**Published:** 2021-05-19

**Authors:** Ariana Hudiță, Ionuț Cristian Radu, Cătălin Zaharia, Andreea Cristina Ion, Octav Ginghină, Bianca Gălățeanu, Luminița Măruțescu, Florin Grama, Aristidis Tsatsakis, Leonid Gurevich, Marieta Costache

**Affiliations:** 1 Department of Biochemistry and Molecular Biology, University of Bucharest, 91–95 Splaiul Independentei Street, 050095 Bucharest, Romania; ariana.hudita@bio.unibuc.ro (A.H.); marieta.costache@bio.unibuc.ro (M.C.); 2 Advanced Polymer Materials Group, University Politehnica of Bucharest, 1–7 Gh. Polizu Street, 011061 Bucharest, Romania; ionut.radu@upb.ro (I.C.R.); catalin.zaharia@upb.ro (C.Z.); l.andreeacristina@yahoo.com (A.C.I.); 3 Department of Surgery, “Sf. Ioan” Emergency Clinical Hospital, 13 Vitan Barzesti Street, 042122 Bucharest, Romania; octav.ginghina@umfcd.ro; 4 Department II, Faculty of Dental Medicine, “Carol Davila” University of Medicine and Pharmacy Bucharest, 17–21 Calea Plevnei Street, 010232 Bucharest, Romania; 5 Department of Microbiology and Immunology, University of Bucharest, 1–3 Aleea Portocalelor Street, 060101 Bucharest, Romania; luminita.marutescu@bio.unibuc.ro; 6 General Surgery Department, Coltea Clinical Hospital, 1 I.C. Brătianu Street, District 3, 030171 Bucharest, Romania; florin_gramma@yahoo.com; 7 Department of Toxicology and Forensic Sciences, Faculty of Medicine, University of Crete, 71003 Heraklion, Greece; tsatsaka@uoc.gr; 8 Department of Materials & Production, Aalborg University, Skjernvej 4A, 9220 Aalborg, Denmark; lg@mp.aau.dk

**Keywords:** 5FU, polymeric nanoparticles, drugs delivery systems, colorectal cancer, cytotoxicity, inflammation, migration and invasion

## Abstract

5-fluorouracil (5-FU) remains the gold standard of treatment for colorectal cancer, but its poor bioavailability and high systemic toxicity highlight the urgent need for the development of novel delivery strategies to increase the efficacy of 5-FU treatment. The present study is aimed to design and validate a PEGylated Silk Fibroin Nanocarrier (SF/PEG nanoparticles (NPs)) as an efficient 5-FU delivery system for potential intravenous administration. Using the human adenocarcinoma HT–29 cell line as an in vitro model for colorectal cancer, the cytotoxicity screening of the SF/PEG NPs showed that pristine nanocarriers were highly biocompatible, while the addition of 5-FU triggers a dramatic reduction in tumor cell viability, proliferation potential and mitochondrial integrity as well as a significant increase in nitric oxide production. Despite their high in vitro cytotoxicity, the 5-FU SF/PEG NPs were found hemocompatible as no impact on red blood cells hemolysis or the phagocytic activity of the granulocytes was observed. Exposure of HT–29 tumor cells and blood samples to 5-FU SF/PEG NPs augmented the tumor necrosis factor-α levels. Moreover, 5-FU SF/PEG NPs showed an impact on tumor cell migration and invasive potential as both of these processes were inhibited by the NP treatment.

## 1. Introduction

Colorectal cancer (CRC) is the third most common type of cancer and the first cause of death among gastrointestinal cancers worldwide [1,2]. The conventional therapeutic approach mainly involves surgery followed by chemotherapy and is dependent on the disease stage and the tumor location [3,4,5]. 5-fluorouracil (5-FU), as well as its prodrug capecitabine, are used as first-line drugs for various chemotherapy regimens in CRC treatment [6]. Being a part of the antimetabolite drugs family, 5-FU interferes with key biosynthetic processes and is also incorporated into nucleic acids inhibiting their normal function [7]. However, its administration alone produces an initial response of only 40–50% [8]. On the other hand, the biochemical breakdown of 5-FU is directly dependent on the rate-limiting catabolic enzyme called dihydropyrimidine dehydrogenase (DPD), which is encoded by the *DPYD* gene. Considering that DPD converts approximately 80–85% of 5-FU into its inactive metabolite [9] and that decreased DPD activity leads to less drug clearance [10], the presence of *DPYD* genotypic variants is a key factor in 5-FU systemic toxicity and drug resistance [11,12]. Aberrant expression of thymidine synthetase and/or p53 mutations are also believed to contribute to 5-FU resistance [13,14]. Numerous studies have investigated potential approaches to decrease the toxicity of conventional antineoplastic drugs and increase the chemical sensitivity to achieve better curative effects of chemotherapy and gain more benefits for patients with CRC [15,16]. 5-FU has been studied for its potential to induce apoptosis by triggering several signaling pathways. Tumor necrosis factor α (TNF–α) plays a key role as an inflammatory mediator with a high impact on carcinogenesis, due to its participation in chronic inflammatory diseases [17,18], apoptosis, cell proliferation, and differentiation. However, the role of TNF–α in cancer remains controversial. Some studies proved that elevated concentrations of TNF–α induce an antitumoral response in a murine model of sarcoma [19]. In contrast, other studies reveal that a decreased production of TNF–α induces a tumor phenotype [20]. Lastly, the tumorigenesis of TNF–α might be based on its ability to stimulate reactive oxygen species (ROS) and reactive nitrogen species (RNS) production, inducing DNA damage [21,22].

5-FU exposure to DPD in the bloodstream, drug resistance, as well as systemic toxicity associated with its administration, are key challenges in the current chemotherapy of CRC, and therefore, new targeted approaches are required to improve the overall therapeutic outcome in CRC. In this respect, nanomedicine has gained much interest in the past few years in oncology due to a large variety of nanocarriers developed both for targeted delivery of drugs with low bioavailability and high toxicity [23,24,25,26] and for protecting drugs against degradation before reaching the final target. Polymeric nanoparticles in particular are a versatile choice for the delivery of both hydrophilic and hydrophobic drugs [27,28] or other bioactive molecules [29,30]. Silk fibroin has been previously proved as a biocompatible material, suitable for the development of 5-FU delivery nanoshuttles in colorectal cancer [31]. 

In this context, the aim of the current study was to validate a PEGylated silk fibroin (SF/PEG) nanocarrier as an efficient 5-FU delivery system with potential intravenous administration. For this, we prepared pristine and 5-FU loaded nanocarriers and characterized them in terms of morphology and drug release potential. Furthermore, we determined the in vitro working dose on HT–29 adenocarcinoma cells and validated the nanoparticles’ internalization potential to sustain the cytotoxicity results. The cytotoxic potential of the nanocarriers was investigated both on colorectal adenocarcinoma cells and blood cells. Lastly, the migration and invasiveness of the cancer cells were evaluated under the treatment with the proposed 5-FU drug delivery system.

## 2. Materials and Methods

Materials: Bombyx mori cocoons were kindly provided by SC Sericarom SA, sodium carbonate (Na_2_CO_3_) and sodium bicarbonate (NaHCO_3_) by Chimopar Trading SRL, sodium dodecyl sulfate was obtained from Merck (Kenilworth, NJ, USA) and lithium bromide (LiBr) was purchased from Honeywell (Charlotte, CA, USA). Acrylic acid, tetra(ethylene glycol) diacrylate (PEG diacrylate) with an average molecular weight of 302 g/mol and potassium persulfate (KP) were purchased from Sigma-Aldrich (St. Louis, MO, USA).

### 2.1. 5-FU Loaded PEGylated Silk Fibroin Nanoparticles Preparations and Characterization

#### 2.1.1. Production of Pristine and 5-Fluorouracil Loaded Polymeric Nanocarriers 

The polymeric nanocarriers were produced from natural silk fibroin (SF) modified with acrylic acid (SF-Ac) and tetra(ethylene glycol) diacrylate (PEG). Briefly, acrylic acid was added to 5 wt.% SF solution in a ratio of 1:100, *v/v* acrylic acid to fibroin solution. The modified SF was dialyzed for one week against distilled water, and then it was washed on filter paper. A dialysis membrane with MWCO of 14 kDa was used. Next, it was solubilized in 10 M lithium bromide aqueous solution resulting in 5 wt.% SF-Ac solutions. The obtained SF-Ac solution was mixed with 10 wt.% aqueous PEG solution in a volumetric ratio of 1:10 (SF-Ac:PEG solutions). A potassium persulfate initiator was added to the mixture and incubated at 60 °C for 5 h. The obtained nanoparticles (NPs) were recovered by filtration. For fluorescent NPs, fluorescein isothiocyanate (FITC) was added to the final reaction mixture (6 mg FITC/1 g NPs) and incubated for 24 h.

The loading of the 5-fluorouracil (5-FU) drug was done by an indirect method. The polymeric NPs were immersed in an aqueous solution of 5-FU (1 mg/mL) for 24 h. The obtained 5-FU SF/PEG NPs were dried in an oven at 50 °C. For 5-FU release studies, 5 mL of drug-loaded nanoparticles (3 wt.%) was placed into a cellulose dialysis tube, which was then immersed in 35 mL of phosphate-buffered saline (PBS) at pH 7.45. Samples were collected every 15 min during the first hour and then every 30 min and analyzed by UV-VIS spectroscopy (UV-3600 Shimadzu UV-VIS-NIR spectrophotometer). To maintain a constant volume, after each collection, 5 mL of fresh PBS was added to the flask. Each experiment was repeated three times.

#### 2.1.2. Characterization of the SF/PEG Nanoparticles

SF/PEG nanoparticles ((SF/PEG NPs) were visualized using a Quanta Inspect F Scanning Electron Microscope (FEI Company) equipped with a field emission gun with 1.2 nm resolution and an X-ray energy dispersive spectrometer (EDS) after coating with a thin layer of gold.

The size distribution of PEGylated silk fibroin nanoparticles in solution was evaluated by Dynamic Light Scattering (DLS) using a Malvern Zetasizer Nano (model ZEN5600, Malvern, UK). The same instrument was used for measuring of zeta-potential of SF/PEG NPs. 

### 2.2. Cell Culture and Experimental Design

Human adenocarcinoma HT–29 (ATCC^®^ HTB-38™) cells were cultured in Dulbecco’s Modified Eagle’s Medium (DMEM), supplemented with 10% fetal bovine serum (FBS), 100 U/mL penicillin, and 100 μg/mL streptomycin under humidified air at standard cell culture conditions (37 °C, 5% CO_2_). The culture medium was renewed every other day. For cell culture propagation, HT–29 cells were enzymatically detached from the culture vessels with trypsin/EDTA and subcultivated at a 1:4 ratio. 

### 2.3. Inhibitory Concentration 50 Determination (IC_50_)

To investigate the cytotoxicity of different concentrations of 5-FU PEGylated SF NPs and to determine the optimal working dose for further biological investigations, the MTT viability assay was used. Briefly, HT–29 cells were seeded at an initial density of 5 × 10^3^ cells/well in 96-well culture plates and incubated overnight to allow cellular attachment. The next day, the culture medium was discarded and replaced with the following dilutions of a 100 mg/mL 5-FU PEGylated SF NP stock solution, freshly prepared: 20 mg/mL, 15 mg/mL, 12 mg/mL, 10 mg/mL, 8 mg/mL, 6 mg/mL, 4 mg/mL and 2 mg/mL. For the untreated experimental controls, the culture medium was refreshed. After 24 h of treatment, all the culture media were discarded and replaced with 1 mg/mL 3-(4, 5-dimethyldiazol-2-yl)-2, 5-diphenyltetrazolium bromide MTT solution (Sigma Aldrich, St. Louis, MO, USA), freshly prepared in FBS free culture medium. Following 3 h of incubation at 37 °C, the MTT solution was removed and the resulting formazan crystals were solubilized in DMSO. The optical density (OD) of the resulting solution was measured at 550 nm using a FlexStation III microplate multimodal reader (Molecular Devices). After subtracting the background control for all the measurements, the cell viability (%) was expressed as a percentage with respect to the untreated control cells by using the following formula: (% cell viability) = [100 × (sample abs)/(control abs)]. The IC_50_ was determined as the concentration at which the 5-FU PEGylated SF NPs induced a 50% reduction in cell viability as compared with the untreated control. 

### 2.4. In Vitro Cytotoxicity Screening 

Before the in vitro biological investigations, the capacity of HT–29 cells to internalize the novel synthesized 5-FU drug delivery systems was assessed by flow cytometry. Briefly, HT–29 cells were seeded in 6 well plates at an initial density of 1 × 10^6^ cells per well and treated with 12 mg/mL FITC–labeled SF/PEG NPs. After 24 h, the media was discarded and the cell monolayers were washed 3 times with PBS to remove non-internalized particles. Afterward, the cells were trypsinized, resuspended in 300 μL of PBS, and analyzed by flow cytometry (Cytoflex, Beckman Coulter, Brea, CA, USA). Data acquisition and analysis were carried out using Beckman Coulter CytExpert Data. 

To investigate the cytotoxic potential of the 5-FU PEGylated SF nanocarriers, HT–29 cells were seeded at an initial density of 1 × 10^4^ cells/well in 96–well plates for all spectrophotometric assays and 1 × 10^5^ cells/well in 12 -well plates for all microscopy-based assays. Then, 24 h post-seeding, the culture medium was discarded and replaced with 12 mg/mL solutions of pristine and 5-FU SF/PEG NPs in the culture medium. The nanoparticle working solutions were freshly prepared in a complete culture medium right before treatment. After 24 h and 72 h of treatment, the following assays were performed to characterize the in vitro cytotoxic potential of the 5FU PEGylated SF NPs:

#### 2.4.1. MTT Viability Assay

The effect of pristine and 5-FU loaded SF/PEG NPs on HT–29 cell viability was investigated using the MTT assay. Briefly, 24 h and 72 h post-treatment, the media was discarded and the MTT assay was performed as described above. The results were expressed as the percentage of viable cells relative to untreated control cells.

#### 2.4.2. Mitotracker Assay

The mitochondrial integrity of the HT–29 cells exposed to pristine and 5-FU loaded PEGylated SF NPs was evaluated after MitoTracker™ Red CMXRos (Thermo Fisher Scientific, Waltham, MA, USA) staining, according to the manufacturer’s recommendation. At each experimental time, the culture medium and treatment solutions were removed and replaced with a prewarmed staining solution containing 100 nM Mitotracker. After 40 min of incubation in standard cell culture conditions, the staining solution was removed and cells were fixed with 4% paraformaldehyde (PFA) solution and permeabilized using a 2% bovine serum albumin (BSA)/0.1% Triton X-100 solution. Prior to imaging, the nuclei of HT-29 tumor cells were stained with 4′,6-diamidino-2- phenylindole (DAPI). The HT-29 monolayers were analyzed using the IX73 Olympus fluorescence microscope and CellSenseF software.

#### 2.4.3. Live and Dead Assay

The effect of 5-FU loaded SF/PEG NPs on HT-29 cell survival was evaluated using the Live/Dead kit (Invitrogen). After 24 h and 72 h of treatment, the HT—29 monolayers were washed with PBS and stained with a fresh solution containing 2 μM calcein AM and 4 μM EthD-1 available in the staining kit. After 15 min of incubation at room temperature in the dark, the staining solution was replaced with PBS and the probes were investigated using an IX73 Olympus fluorescence microscope and CellSenseF software. 

#### 2.4.4. LDH Cytotoxicity Assay

The potential of pristine and 5-FU loaded SF/PEG NPs to induce lesions on the HT–29 cell membrane was investigated by measuring the levels of lactate dehydrogenase (LDH) released in the cell culture medium by injured cells. For this, media samples were harvested from HT–29 control cells or HT–29 cells exposed to the treatment and processed with a TOX7 lactate dehydrogenase in vitro using the toxicology kit according to the manufacturer guidelines. The optical density (OD) of the final solutions was measured at 490 nm using a microplate multimodal reader.

#### 2.4.5. Nitric Oxide Assay

The production of nitric oxide (NO) by tumor HT–29 cells as a response to pristine and 5-FU loaded SF/PEG NP treatment was measured using Griess Reagent (Promega, Madison, WI, USA). Briefly, after 24 h and 72 h of treatment, the culture medium was harvested and 50 μL of culture supernatants were mixed with 50 μL Sulfanilamide Solution and incubated at room temperature in the darkness for 20 min. At half the incubation time, 50 μL of N-1-naphthyl ethylenediamine dihydrochloride (NED) solution was added. The optical density (OD) of the resulting solutions was read at 550 nm using the multimodal reader Flex Station III. The concentration of NO was extrapolated from a nitrite standard reference curve that was prepared according to the instructions provided in the kit.

### 2.5. Migration and Invasion

The effect of pristine and 5-FU SF/PEG NP treatment on the migrative and invasive potential of tumor HT–29 cells was assessed using transwell chambers (8 μm, Corning, New York, NY, USA). For the migration assay, HT-29 cells were plated at a density of 5 × 10^4^ cells/well into the upper chamber with FBS—free medium, or with FBS—free medium with 12 mg/mL NPs. The lower chamber was filled with 20% FBS supplemented serum. After incubation with the NPs for 48 h, the upper surface of the inserts was removed using a cotton swab, and then the inserts were fixed with 4% PFA and stained with 0.5% crystal violet solution. Following imaging of the inserts with contrast phase microscopy, the migrative HT—29 cells were destained on a shaker with a 10% acetic acid solution for 20 min, and finally, the optical density of the resulting solutions was measured at 590 nm with a microplate reader. For the invasion assay, the same experimental protocol was used with the only difference that the upper chamber was precoated with Matrigel prior HT—29 cells seeding. After subtracting the background control for all measurements, the migration/invasion (%) was expressed as a percentage to the untreated control cells by using the following formula: (% migration/invasion) = [100 × (sample abs)/(control abs)].

### 2.6. Investigation of TNF–A Protein Expression 

Blood levels of TNF–α as well as the expression of TNF–α by HT-29 cells were investigated under the treatment with pristine and 5-FU loaded SF/PEG NPs by a flow cytometry bead-based assay, using Human Inflammatory Cytokine Cytometric Bead Array (CBA) (Becton Dickinson) kit. For this, complete culture media were harvested from HT–29 cultures after 24 h and 72 h of treatment with pristine and 5-FU loaded SF/PEG NPs. At the same time points, culture media were harvested from untreated cells to serve as a reference. According to the manufacturer’s recommendations, 50 μL of the sample was incubated for 2 h at room temperature and darkness with 50 μL of TNF–α Capture Beads and 50 μL Inflammation PE Detection Reagent. The TNF–α levels in the blood were quantified after 6 h and 24 h of whole blood samples incubation with 12 mg/mL pristine and 5-FU loaded SF/PEG NPs using the same bead-based assay and protocol. After a wash step, all tubes were analyzed in a Cytoflex (Beckman Coulter, Brea, CA, USA) flow cytometer using CytExpert Data for sample acquisition and data analysis. The results were graphically represented and statistically analyzed with GraphPad Prism Software.

### 2.7. Blood Interaction with 5-FU PEGylated Silk Fibroin Nanoparticles

Peripheral blood samples from volunteer healthy donors were collected using EDTA or sodium citrate blood collection vacutainers (Becton Dickinson) after obtaining their informed consent. 

#### 2.7.1. Red Blood Cell Assay

To evaluate the potential lytic effect of pristine and 5-FU SF/PEG NPs on red blood cells, EDTA–blood samples were used. Briefly, blood samples were centrifuged and the erythrocytes recovered and washed in 150 mM NaCl. The obtained pellet was resuspended in PBS to reach a final volume of 5 mL. The obtained erythrocytes solution was further diluted 1:50 with PBS and 190 μL of this solution was distributed in 96–well round bottom plates. A volume of 10 μL of the following solutions was added to each well, in triplicate: PBS for negative controls, 20% Triton X-100 for positive controls, and 12 mg/mL of pristine and 5-FU loaded SF/PEG NPs solutions. The samples were incubated for 1 h at 37 °C on a plate shaker and centrifuged. The resulting supernatants were transferred to a new 96–well plate and the optical density (OD) of these solutions was measured at 492 nm. The following formula was used for expressing hemolysis as a percentage: (% hemolysis) = 100 × [(sample abs–negative control abs)/(positive control abs–negative control abs)].

#### 2.7.2. Phagocytic Activity of Granulocytes

Sodium citrate-blood samples were used to investigate the phagocytic activity of granulocytes in human whole blood. The oxidative burst of granulocytes stimulated with *E. coli* and exposed to pristine and 5-FU loaded SF/PEG NPs was investigated using the FagoFlexEx^®^ kit (Exbio, Vestec (Prague-West District), Czech Republic) kit and flow cytometry. A volume of 50 µL of heparinized whole blood was mixed gently with 10 µL PBS (negative control) or *E. coli* (as positive control) and with 10 μL dihydrorhodamine-123 (DHR123) and incubated in a water bath (20 min at 37 °C). The influence of the tested NPs on the phagocytic activity of the granulocytes was determined by the addition of 12 mg/mL pristine and 5-FU loaded SF/PEG NPs to this mix. After incubation, the erythrocytes were lysed at room temperature for 10 min with 1 mL of FACS lysis solution. The fluorescence intensity of rhodamine 123, produced by DHR123 oxidation, was detected by flow cytometry on Accuri C6 Plus (BD Biosciences, Franklin Lakes, NJ, USA) cytometer. The results obtained were further analyzed with the instrument’s software and shown as a percentage of rhodamine positive granulocytes gated on the side-scatter and forward-scatter dot plot to exclude the debris and cellular aggregates.

### 2.8. Statistical Analysis 

Statistical analyses were carried out using GraphPad Prism Software. All statistic data are presented as mean values ± standard deviation of three independent experiments. Both one- and two-way analyses of variance (ANOVA) were used. Bonferroni’s multiple comparisons posttest were used to identify which groups were different, with *p* < 0.05 considered statistically significant. 

## 3. Results and Discussion

### 3.1. PEGylated Silk Fibroin Nanoparticle Synthesis and Characterization

Following the experimental protocols described above, the following types of PEGylated SF NPs were obtained: pristine NPs, 5-FU loaded NPs, and FITC–labeled NPs. The nanocarriers exhibit semi-interpenetrating cross-linked network structures, a characteristic that ensures chemical interaction between the natural and synthetic polymers for a controlled drug release.

**Figure 1:**
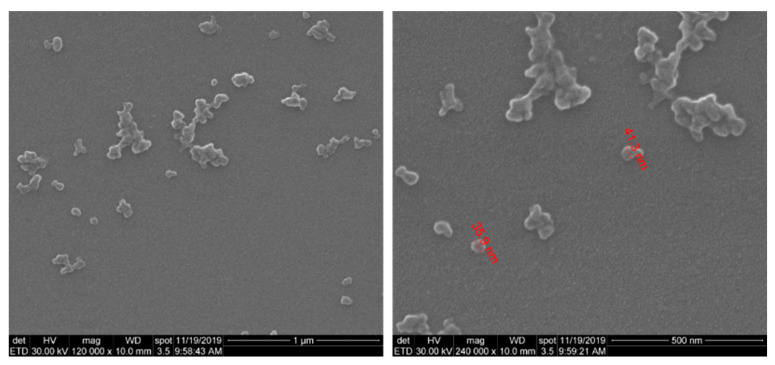
SEM microphotographs of SF/PEG nanoparticles.

The SEM morphological investigation of SF/PEG NPs revealed that nano-sized particles with dimensions below 50 nm were obtained (Figure 1). Besides individual nanoparticles, larger aggregates were also observed. Moreover, SEM images revealed a clean surface of SF/PEG NPs, with no traces of residual mass.

**Figure 2:**
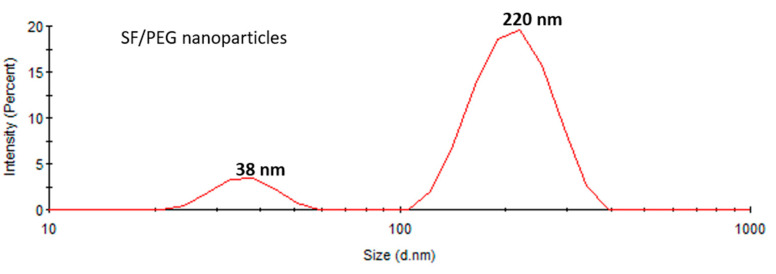
Size distribution of SF/PEG pristine nanoparticles obtained by DLS.

The size distribution of SF/PEG NPs in an aqueous solution was investigated by DLS. The size distribution profile of the pristine particles revealed two peaks (Figure 2), one with a mean size of around 38 ± 2 nm and the other with a mean size of 220 ± 10 nm with a polydispersity index (PDI) of 0.33 fitting a polydisperse bimodal model. These results are in good agreement with the SEM data, showing both small individual nanoparticles and aggregates. The zeta potential of SF/PEG NPs was found to be −7.73 ± 0.8 mV.

**Figure 3:**
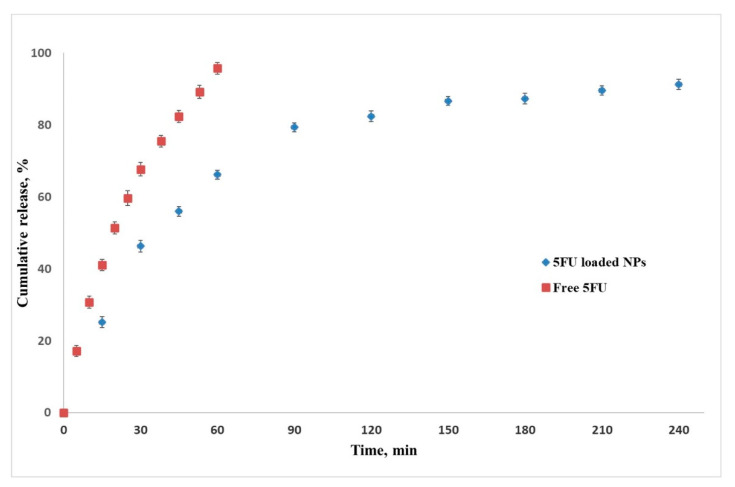
5-fluorouracil release profile from SF/PEG NPs. Data shown as the mean ± SD (n = 3).

The drug loading ability of this nanoparticle formulation was found to be high, with a drug loading efficiency of 93% ± 3. UV–VIS data analysis showed a rapid release of 5-FU from the SF/PEG NPs during the first 60 min (65% cumulative release), followed by a slower release trend for the next 180 min (89% cumulative release), as shown in Figure 3. The rapid release of the loaded 5-FU is favored by the high swelling ability of the cross-linked structure. In this way, the liquid medium penetrates the nanoparticles and the drug is released [32]. Free 5-FU is rapidly released within 60 min (98% cumulative release), therefore, from the above results, it was found that the nanoparticles had the effect of prolonging the drug release compared with the 5-FU solution.

### 3.2. Inhibitory Concentration 50 Determination (IC_50_)

**Figure 4:**
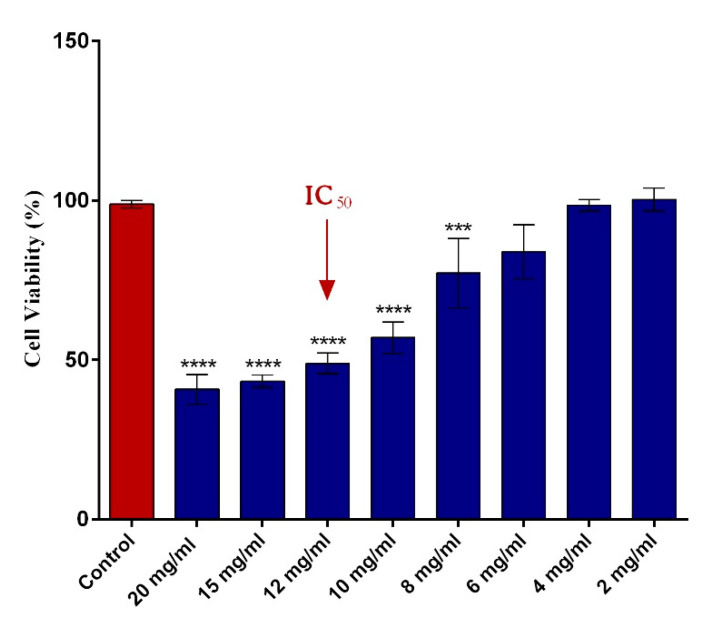
Human adenocarcinoma HT–29 cell viability after 24 h incubation with various concentrations (2–20 mg/mL) of 5-FU PEGylated SF NPs (**** *p* ≤ 0.0001 sample vs. untreated control; *** *p* ≤ 0.001 sample vs. untreated control). The red arrow indicates the 5-FU SF/PEG NPs concentration corresponding to the IC_50_.

The impact of different concentrations of 5-FU PEGylated SF NPs on HT–29 cellular viability was assessed by the MTT assay. As presented in Figure 4, the cell viability of HT–29 tumor cells decreased in a dose-dependent manner with the concentration of the 5-FU SF/PEG NPs applied. While low concentrations (2–6 mg/mL) did not impact the viability of HT–29 cells, a significant decrease of the viable HT–29 tumor cells was observed starting with 8 mg/mL of 5-FU SF/PEG NPs. The analysis of the obtained data allowed the IC_50_ determination for 5-FU SF/PEG NPs. A 50% decrease of HT–29 cell viability was observed following 24 h exposure to 12 mg/mL 5-FU SF/PEG NP treatment. Therefore, the 12 mg/mL concentration was considered the IC_50_ and was used as the experimental working dose for all further biological investigations. 

### 3.3. Basic In Vitro Cytotoxicity Screening of 5-FU PEGylated Silk Fibroin Nanoparticles

The cytotoxic potential of the original 5-FU loaded nanocarriers was investigated after 24 h and 72 h exposure of HT–29 cells to 12 mg/mL 5-FU SF/PEG NPs. To assess whether potential cellular damage occurs in response to the loaded drug or the nanocarrier itself, HT–29 cells were also exposed to 12 mg/mL pristine SF/PEG NPs for the same period. 

First, the FITC labelling of the SF/PEG NPs allowed the investigation of the HT–29 cellular uptake of SF/PEG NPs by flow cytometry. The obtained results showed that after 24 h of treatment, 99.08% ± 0.06 of the tumor cells showed FITC signal indicating efficient nanoparticle uptake by HT–29 cells (data not shown). 

**Figure 5:**
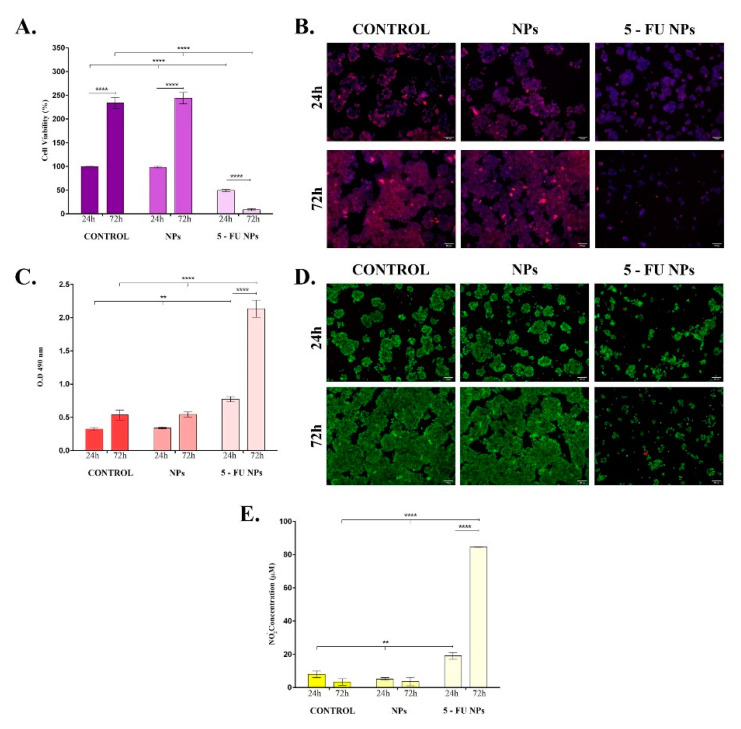
In vitro basic cytotoxicity screening of simple and 5-FU PEGylated SF NPs. The cytotoxicity of the pristine and 5-FU loaded SF/PEG NPs (12 mg/mL) was investigated after 24 h and 72 h of treatment in terms of: (**A**). Cell viability; (**B**). Mitochondrial integrity; (**C**). LDH release; (**D**). Cell viability and proliferation potential; (**E**). NO release (** *p* ≤ 0.01; **** *p* ≤ 0.0001). The fluorescence micrographs were obtained by after labelling HT–29 cells with Mitotracker and DAPI (**B**) or calceinAM and EthBr (**D**).

Investigation of SF/PEG NPs ± 5-FU treatment impact on HT–29 cellular viability showed that the cell metabolic activity was severely altered only by 5-FU loaded nanoparticles (Figure 5A). HT–29 cell cultures exposed to pristine SF/PEG NPs exhibited a similar ratio of metabolically active cells to the untreated HT–29 cell cultures at both experimental time points, showing that the polymeric nature of the nanocarrier and PEG addition did not affect the tumor cell viability. In contrast, the HT–29 cell viability was significantly (*p* ≤ 0.0001) decreased upon the treatment with 5-FU PEGylated SF NPs; only <10% of the treated HT–29 cells were viable after 72 h of treatment. The HT–29 proliferative status was sustained by bare SF/PEG NPs as highlighted by the 2.5-fold increase of the cell viability between 24 h and 72 h of culture (*p* ≤ 0.0001).

Furthermore, the HT–29 tumor cells were stained with the mitochondrial potential-independent Mitotracker dye to label the existing live mitochondria within HT–29 cell cultures exposed for 24 h and 72 h to pristine and 5-FU labelled SF/PEG NPs (Figure 5B). Untreated HT–29 tumor cells and pristine SF/PEG NP-treated HT–29 tumor cells were viable after 24 h and 72 h of cultures, showing a strong positive signal for the mitochondrial staining. The addition of 5-FU in the SF/PEG NPs triggered a dramatic decrease of the mitochondrial membrane potential after 24 h of treatment. Moreover, after 72 h of treatment, an extremely weak positive signal for the Mitotracker staining was identified in HT–29 cells treated with 5-FU loaded NPs, showing that prolonged exposure to the loaded nanocarriers severely affects the tumor HT–29 cells viability and mitochondrial integrity. 

The ability of the SF/PEG NPs ± 5-FU NPs to affect the HT–29 cell membrane integrity was investigated by measuring the extracellular LDH concentrations after 24 h and 72 h of treatment (Figure 5C). No significant differences in the LDH levels were observed between SF/PEG NPs-treated and untreated HT–29 cells at any of the investigated time points, showing that the pristine nanocarrier does not alter the HT–29 cell membrane integrity. In contrast, the 5-FU NP treatment induced a significant increase of the leaked LDH, showing that the drug-loaded nanocarriers induce lesions in the HT–29 cell structure. The detected levels of LDH in HT–29 cell cultures under 5-FU SF/PEG NP treatment were significantly higher after both 24 h (*p* ≤ 0.01) and 72 h (*p* ≤ 0.0001) of exposure as compared with untreated control cultures. After 72 h, the LDH levels detected in the culture medium of HT–29 cells exposed to 5-FU SF/PEG NPs were significantly (*p* ≤ 0.0001) increased in comparison with 24 h levels, showing that the 5-FU-loaded NPs alters the cell membrane integrity of HT–29 cells in a time-dependent manner. 

Our findings regarding the impact of SF/PEG NPs ± 5-FU treatment on HT–29 cell viability and proliferation potential were confirmed by Live/Dead assay (Figure 5D). The fluorescence microscopy investigation of HT–29 cell cultures showed that HT–29 cells proliferated both in the untreated cultures as well as in the samples treated with pristine SF/PEG NPs. More, cell viability between these two samples was similar during the experimental time frame as no dead red-stained cells were identified in none of the cultures. In contrast, when treated with 5FU loaded SF/PEG NPs, HT—29 cells viability and proliferation potential dramatically decreased. Moreover, the 5-FU NP treatment was found to affect the ability of HT–29 to form cell clusters as highlighted by the different cell organization patterns revealed in 5-FU NP-treated cultures as compared with the control and pristine NPs-treated cells. 

Finally, the ability of SF/PEG ± 5-FU nanocarriers to enhance nitric oxide (NO) production was evaluated by measuring the nitrite levels in HT–29 cell cultures exposed for 24 h and 72 h to pristine and 5-FU loaded NPs (Figure 5E). The pristine SF/PEG NPs did not enhance NO production as no modifications of the nitrite levels were detected after 24 h and 72 h of treatment between controls and treated samples. The 5-FU SF/PEG NPs induced a significant (*p* ≤ 0.01) enhancement of the nitrite production as the concentration detected after 24 h of treatment was twice the concentration detected in untreated samples. Moreover, after 72 h, only low concentrations of nitrite (3 μM) were detected in untreated and pristine NP-treated HT–29 cell cultures, in contrast with the 5-FU NP-treated HT–29 cells where the nitrite concentrations were higher than 80 μM (*p* ≤ 0.0001).

Due to its excellent biocompatibility and biodegradability, SF has become a popular choice for drug delivery systems development [33]. SF–based nanocarriers demonstrate a superior entrapment of 5-FU compared to other polymeric NPs due to the hydrophobic amino acid residues present in the SF molecules [34]. Furthermore, NP PEGylation strategy can increase the NP stability and biocompatibility and trigger an enhanced accumulation of the drug delivery systems in the tumor tissue [35,36]. These observations are in line with our basic cytotoxicity screening of the original PEGylated SF nanocarriers that revealed that the unloaded nanoparticles are highly biocompatible with the HT–29 tumor cells as no alterations of cell viability, proliferation potential, and mitochondrial integrity were observed upon cells exposure to SF/PEG NPs. 

In contrast, the release of the entrapped 5-FU from the SF/PEG NPs severely altered the investigated biological aspects. The use of drug delivery systems for 5-FU transport to the colorectal cancer tumor cells is a modern strategy to address critical issues identified for the administration of free 5-FU such as short biological half-life, poor distribution to the tumor tissue, and severe toxic effects [37]. The 5-FU loaded SF/PEG NPs exhibited high cytotoxicity and antiproliferative activity and severely decreased the cell viability of HT–29 tumor cells. A decrease in the HT–29 cell viability was also observed by Rahmani and colleagues after biocompatible 5-FU loaded SF NP treatment [38]. Moreover, the 5-FU release from the SF/PEG nanosystems triggered an enhancement of the NO production in HT–29 cell cultures. While low concentrations of NO promote tumor growth, high concentrations of NO promote tumor cell apoptosis [39]. In this context, the elevated levels of NO detected in the HT–29 cultures could be responsible for the dramatic decrease of viable HT–29 tumor cells following 5-FU SF/PEG NP treatment. 

### 3.4. Migration and Invasiveness Potential Alterations

**Figure 6:**
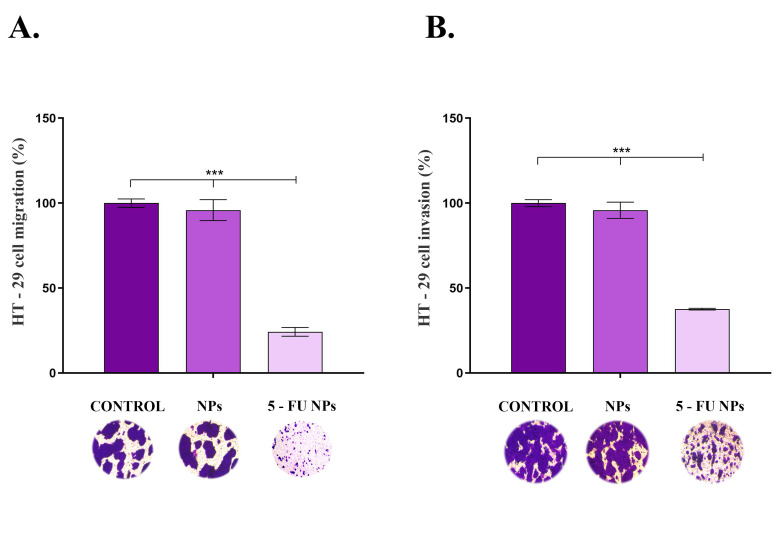
HT–29 (**A**). cell migration and (**B**). invasion potential after 48 h of exposure to pristine and 5-FU loaded PEGylated SF NPs as revealed by transwell-based assays (*** *p* ≤ 0.001). Representative optical microscopy images of HT–29 Crystal Violet-stained cells are shown below each sample.

As cancer cell migration and invasion are involved in colorectal cancer progression and metastasis, the impact of SF/PEG ± 5-FU NPs on HT–29 cell motility was assessed using transwell-based assays (Figure 6). Exposure of HT–29 tumor cells to pristine SF/PEG NPs for 48 h presented no effect on the tumor cell migration (Figure 6A) as the ratio of treated migrative cells was similar to the ratio of untreated migrative cells. However, exposure of HT–29 cells to 5-FU loaded NPs triggered a dramatic decrease in the migrative ability of the tumor cells as revealed by the 75% reduction of the cell migration under this experimental condition as compared with the untreated control (*p* ≤ 0.001). 

The same trend was observed in the invasion assay (Figure 6B), where the HT–29 tumor cells’ invasive potential was severely altered upon exposure to 5-FU loaded PEGylated SF NPs (*p* ≤ 0.001), while no impact on HT–29 invasiveness was observed under treatment with pristine SF/PEG NPs. As colorectal cancer presents a rapid progression, the ability of the novel 5-FU loaded NPs to inhibit HT–29 tumor cells migration and invasion potential is an essential feature for the prospective use of these drug–delivery cancer systems in CRC treatment. 

### 3.5. Modulation of TNF–A Expression under the Treatment with 5-FU PEGylated Silk Fibroin Nanoparticles

TNF–α is a pro-inflammatory cytokine produced both by macrophages and tumor cells [40]. A quick review of the literature shows that TNF–α plays key roles in inflammation (including the promotion of tumoral inflammatory processes), apoptosis, cell proliferation, and differentiation. TNF–α displays its anti-tumor effect by acting as an enhancer of vascular permeability [41] that leads to the accumulation of chemotherapeutics agents inside tumors [42,43]. Van Horssen et al., suggest that endothelial cells of the tumor vasculature highly express the tumor necrosis factor receptor-1 (TNFR-1) as compared with the surrounding normal cells, thus allowing specific targeting of tumors by TNF–α. This will promote an increased permeability of the tumor vessels [44]. 

**Figure 7:**
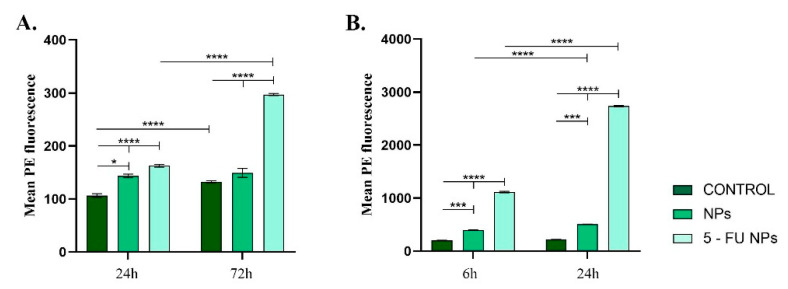
TNF–α expression profile detected in (**A**)**.** HT–29 cell cultures exposed for 24 h and 72 h to 12 mg/mL SF/PEG NPs ± 5-FU and (**B**). whole blood exposed for 6 h and 24 h to 12 mg/mL SF/PEG NPs ± 5-FU (* *p* ≤ 0.05; *** *p* ≤ 0.001; **** *p* ≤ 0.0001).

Both in blood or HT–29 cell cultures, the TNF–α levels varied depending on the NP treatment applied (Figure 7). In vitro, both pristine and 5-FU loaded NPs altered TNF–α expression, showing that independent of the drug load, the empty nanocarrier induced a pro-inflammatory stimulus in the HT–29 cell cultures (Figure 6A). However, the addition of 5-FU to the nanocarrier induced a dramatic enhancement in the TNF–α production (*p* ≤ 0.0001). The increased expression of TNF–α might explain the elevated levels of NO as TNF–α is known to promote cellular oxidative stress [21,22]. A similar pattern of TNF–α production was observed following blood samples incubation with the tested NPs (Figure 6B). After pristine SF/PEG NP treatment, low levels of TNF–α were detected in plasma samples, but the cytokine level was significantly increased when compared to control plasma samples (*p* ≤ 0.001) at both investigated time points. In response to 5-FU loaded NPs, a dramatic enhancement of TNF–α production was noticed as compared to the untreated samples or samples treated with pristine NPs. Prolonged exposure of the blood samples to 5-FU SF/PEG triggered a 2.5-fold increase of the cytokine (*p* ≤ 0.0001). The increased levels of TNF–α could stimulate other blood cells (macrophages, natural killer cells) to produce potent tumor-supressing molecules [45]. 

### 3.6. Hemocompatibility of 5-FU PEGylated Silk Fibroin Nanoparticles 

Considering that the nanocarriers described in this study were designed for intravenous administration, their potential interaction with blood components is essential. In this context, 5-FU SF/PEG interaction with erythrocytes and granulocytes, as well as an inflammatory response triggered by blood–NPs interaction was investigated using human blood samples harvested from healthy donors. 

#### 3.6.1. Hemolytic Activity 

**Figure 8:**
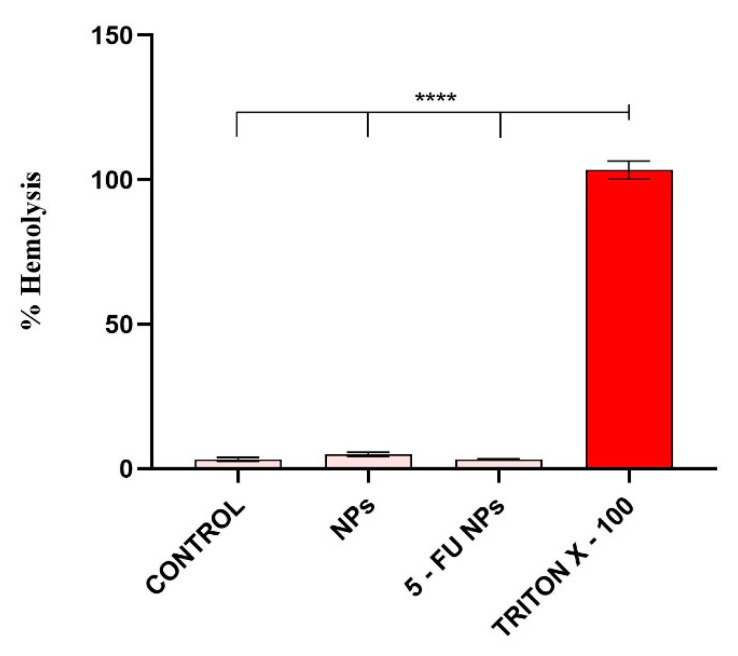
Hemocompatibility of SF/PEG NPs ± 5-FU as revealed by the blood hemolysis assay after incubation with 12 mg/mL SF/PEG NPs ± 5-FU (**** *p* ≤ 0.0001).

In vivo, NPs–induced erythrocyte hemolysis could promote jaundice and anemia [46,47], therefore the hemolytic activity of a novel drug delivery system is an important aspect for its prospective use. The ability of SF/PEG NPs ± 5-FU to damage red blood cell membranes was evaluated using a hemolysis assay based on the spectrophotometric measurement of the hemoglobin released after treatment. The obtained results showed that the SF/PEG NPs do not induce hemolysis, both pristine or 5-FU loaded (Figure 8); the hemolysis level in all treated samples was similar to the untreated control. 

#### 3.6.2. Phagocytic Activity of Granulocytes

**Figure 9:**
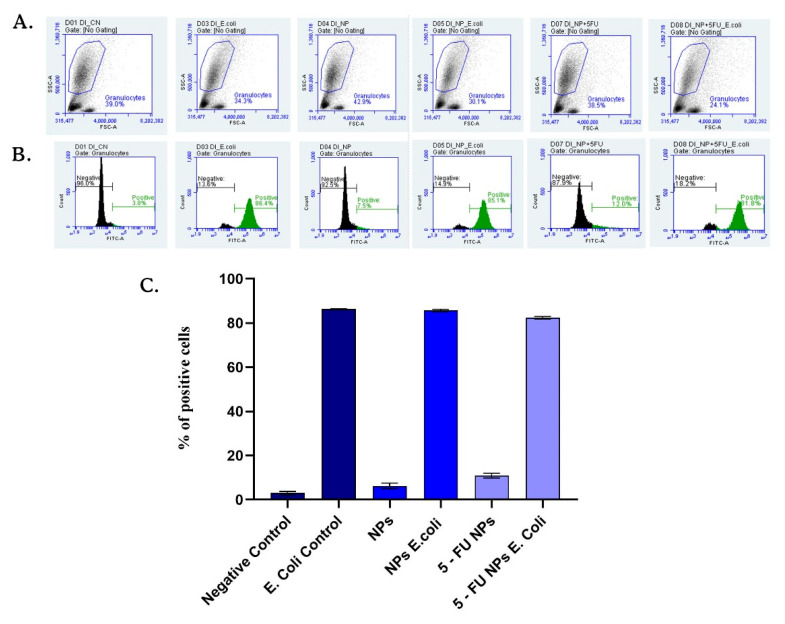
SF/PEG NPs ± 5-FU NPs effect on phagocytic activity of granulocytes. Dot plots (**A**), histograms (**B**), and graphic representation of the mean values of rhodamine-positive granulocytes percentages (**C**) obtained in the presence of tested NPs compared to negative or *E. coli* stimulated controls.

The interaction of granulocytes and SF/PEG NPs ± 5-FU was investigated by assessing the effect of SF/PEG NPs ± 5-FU on the phagocytic activity of blood granulocytes by flow cytometry (Figure 9). The obtained data revealed that the NADPH oxidase of the granulocytes was not activated for producing free radicals by either pristine or 5-FU loaded SF/PEG NPs. After 20 min of incubation of the blood samples with the NPs in absence of *E. coli,* the phagocytosis was not activated as the average values of positive granulocytes were 6.21% for simple NP treatment and 10.8% for 5-FU loaded NP treatment, similar to the values of the negative control. Additionally, the analyzed NPs did not interfere with phagocytosis activation as the results showed that the percentage of phagocyting granulocytes did not change compared to *E. coli* control. Granulocytes are part of the innate immune defense that possess the capacity to recognize foreign materials such as nanoparticles [48,49]. As highlighted by the obtained results, no effect of the SF/PEG NPs ± 5-FU on granulocytes was observed, showing that the investigated NPs do not trigger innate immune system activation. Moreover, the lack of effect of the SF/PEG NPs ± 5-FU on granulocyte phagocytic activity shows that the NPs exhibit lower chances to be engulfed by the granulocytes as a defense clearance mechanism [50]. These aspects could be attributed to the PEGylation, which provides a hydrophilic corona helping NPs to evade immune recognition [51]. 

## 4. Conclusions

In conclusion, nanosized PEGylated Silk Fibroin nanocarriers for intravenous delivery of 5-FU for colorectal cancer therapy were obtained and characterized in vitro. Pristine SF/PEG NPs displayed good biocompatibility on HT–29 colorectal cancer cells and good compatibility with human blood cells. When loaded with 5-FU, the SF/PEG NPs induced a significant decrease in cell viability and increased the expression of TNF-α. Moreover, the migration and invasion potential of the HT–29 adenocarcinoma cells was significantly reduced by the treatment with 5-FU PEGylated Silk Fibroin Nanoparticles. All these findings validate the SF/PEG NP drug delivery system for intravenous administration and pave the way for in vivo studies on animal models, which will reveal the nanocarriers’ bioavailability, elimination route, and potential interactions with vital tissues and organs.

## Data Availability

The data presented in this study are available on request from the corresponding author.
